# Typical magnitude and spatial extent of crowding in autism

**DOI:** 10.1167/16.5.17

**Published:** 2016-03-21

**Authors:** Jan Freyberg, Caroline E. Robertson, Simon Baron-Cohen

**Affiliations:** jf420@cam.ac.uk www.janfreyberg.com; carolinerobertson@fas.harvard.edu http://www.carolineerobertson.com; sb205@cam.ac.uk www.autismresearchcentre.com; Autism Research Centre, Department of Psychiatry, University of Cambridge, Cambridge, UK; Harvard Society of Fellows, Harvard University, Cambridge, MA, USA

**Keywords:** *autism*, *crowding*, *orientation discrimination*, *spatial vision*

## Abstract

Enhanced spatial processing of local visual details has been reported in individuals with autism spectrum conditions (ASC), and crowding is postulated to be a mechanism that may produce this ability. However, evidence for atypical crowding in ASC is mixed, with some studies reporting a complete lack of crowding in autism and others reporting a typical magnitude of crowding between individuals with and without ASC. Here, we aim to disambiguate these conflicting results by testing both the magnitude and the spatial extent of crowding in individuals with ASC (*N* = 25) and age- and IQ-matched controls (*N* = 23) during an orientation discrimination task. We find a strong crowding effect in individuals with and without ASC, which falls off as the distance between target and flanker is increased. Both the magnitude and the spatial range of this effect were comparable between individuals with and without ASC. We also find typical (uncrowded) orientation discrimination thresholds in individuals with ASC. These findings suggest that the spatial extent of crowding is unremarkable in ASC, and is therefore unlikely to account for the visual symptoms reported in individuals with the diagnosis.

## Introduction

The ability to recognize stimuli in our peripheral vision is limited. This ability is hindered by a phenomenon called “crowding,” the breakdown of object recognition in cluttered visual environments (Bouma, 1970). Crowding poses a fundamental limitation on the spatial range over which objects in the periphery of our visual field can be resolved despite accurate detection of their presence (Levi, Hariharan, & Klein, 2002).

Psychophysically, this limitation on perception can be characterized by measuring a participants' “critical distance,” the spatial range over which flankers interfere with target detection. A related measure is the magnitude of crowding, the reduction in visual performance when flankers surround a target at one target–flanker distance relative to when a target is unflanked. These two parameters are influenced by multiple factors, including target eccentricity (Bouma, 1970; Mareschal, Morgan, & Solomon, 2010; Tripathy & Cavanagh, 2002; Westheimer & Truong, 1988), target–flanker feature similarity (Chung, Li, & Levi, 2007; Kennedy & Whitaker, 2010; Kooi, Toet, Tripathy, & Levi, 1994; Louie, Bressler, & Whitney, 2007; Nazir, 1992), and the relative size and position of targets and flankers (Banks, Bachrach, & Larson, 1977; Bouma, 1970; Feng, Jiang, & He, 2007; Toet & Levi, 1992).

Spatial processing of visual information is thought to be atypical in autism spectrum conditions (ASC). Experimentally, people with autism demonstrate superior visual search (O'Riordan, Plaisted, Driver, & Baron-Cohen, 2001; Plaisted, O'Riordan, & Baron-Cohen, 1998), a steeper fall-off in visual acuity with distance from a cued location (Robertson, Kravitz, Freyberg, Baron-Cohen, & Baker, 2013), a bias towards local perception when viewing hierarchical local–global stimuli (Koldewyn, Jiang, Weigelt, & Kanwisher, 2013), and heightened self-reports of visual sensitivity to local details in the visual environment (Robertson & Simmons, 2013).

One component of visual perception that might contribute to autistic visual symptomatology is crowding. Specifically, the reported enhancement of perception of local details in a visual environment may arise from a reduction in peripheral crowding in autism. A few studies have investigated crowding in individuals with ASC, so far with mixed results. One study (Baldassi et al., 2009) tested the effect of crowding on orientation detection , using nine flankers arranged in a circle around a central target at one target–flanker distance (1**°** of visual angle), and found reduced crowding in individuals with ASC. Another study (Kéïta, Mottron, & Bertone, 2010), confirmed this reduced magnitude of crowding in ASC, but only at the closest tested flanker condition (one gap width of a Landoldt C). These findings support the notion that visual processing in ASC may be less vulnerable to crowding effects, specifically within a local spatial region around a visual target. However, two subsequent studies (Constable, Solomon, & Gaigg, 2010; Grubb et al., 2013) reported no differences in the magnitude or the spatial extent of crowding between individuals with and without ASC.

These mixed results may be explained in part by discordance among the paradigms used in previous studies. Crowding is thought to independently occur at multiple levels of visual analysis (Whitney & Levi, 2011): in a given scene, crowding occurs between low-level stimulus features (Parkes et al., 2001), objects parts (Martelli, Majaj, & Pelli, 2005), and whole objects (Farzin, Rivera, & Whitney, 2009). Crowding at these various levels of visual analysis has different characteristics. For example, crowding between simple stimuli with common low-level features (e.g., Gabor patches) is known to lead to compulsory feature-pooling (e.g., orientation; Parkes, Lund, Angelucci, Solomon, & Morgan, 2001). On the other hand, crowding between more complex stimuli such as objects, while driven at least in part by averaging (Dakin, Cass, Greenwood, & Bex, 2010), is also thought to involve probabilistic visual substitution of the flankers and target stimuli (Freeman, Chakravarthi, & Pelli, 2012).

The stimuli used in previous studies of crowding in ASC were of different visual complexities: Baldassi et al. (2009), who found reduced crowding in ASC, used Gabor patches (flanked by vertical Gabors), while Grubb et al. (2013) and Constable et al. (2010), who both found typical crowding in ASC, employed multifeature stimuli (letters and shapes, respectively). This pattern of results may therefore indicate that crowding of simple stimuli is selectively reduced in ASC, but that when multifeature stimuli are used, crowding is intact in ASC. However, two other points of experimental clarification might contribute to the pattern of results. First, the finding of reduced crowding effects in ASC (Baldassi et al., 2009) is difficult to interpret, as the crowding effect in this study was calculated as a ratio between crowded and uncrowded orientation discrimination thresholds, and only the latter was significantly worse in the ASC group. Therefore, their finding of a reduced crowding effect in ASC may have primarily been driven by baseline differences in the groups' orientation discrimination thresholds, rather than crowding per se. Second, only one previous study examined crowding in ASC while monitoring gaze stability (Grubb et al., 2013), leaving open the question of whether previous findings of reduced crowing in ASC might be driven by weaker gaze stability.

We therefore tested the magnitude and spatial extent of crowding in individuals with and without ASC (matched for age, IQ, and gaze-stability) during a feature-based crowding paradigm. Participants performed a simple orientation discrimination task, in which they were asked to identify the tilt of a Gabor target. This paradigm was similar to that used by Baldassi et al. (2009), which demonstrated a striking reduction in crowding in ASC with simple Gabor stimuli. Furthermore, to confirm that our findings were not attributable to basic differences in orientation discrimination between the two groups, we also measured peripheral orientation discrimination without flanking stimuli.

## Methods

### Participants and psychometric testing

Forty-eight participants (25 with ASC) completed Experiments 1–3. Out of these 48, we excluded 13 participants in total from our analyses: six participants (five in the ASC group) based on poor performance during the thresholding procedure prior to the experiment, and seven participants (two in the ASC group) based on poor fixation during the experiment. For details of exclusion criteria, please see *Practice* and *Thresholding* and *Eye tracking* below. It is important to note that excluding participants from the study based on poor performance during the thresholding procedure or poor gaze stability fixation, did not qualitatively affect the results reported here (all *p* > 0.162). Participants were matched for age (Controls: 29.6 ± 8.6, ASC: 33.3 ± 12.3, *p* > 0.31) and nonverbal IQ (Controls: 114.5 ± 22.3, ASC: 121.5 ± 8.8, *p* > 0.22), as assessed using the Wechsler Abbreviated Scale of Intelligence (WASI). ASC participants all had clinical diagnoses of an autism spectrum disorder, as evaluated by qualified clinicians based on DSM-IV criteria, and were assessed using the ADOS-II (Module 4) by a research-reliable experimenter. Participants also completed the Autism Quotient Questionnaire (AQ; Baron-Cohen, Wheelwright, Skinner, Martin, & Clubley, 2001), the Sensory and Perception Questionnaire (SPQ; Tavassoli, Hoekstra, & Baron-Cohen, 2014), and the Glasgow Sensory Questionnaire (GSQ; Robertson & Simmons, 2013). All participants had normal or corrected-to-normal vision, and did not have a diagnosis of epilepsy or attention-deficit / hyperactivity disorder. All participants had normal or corrected-to-normal vision and no diagnosis of epilepsy or attention-deficit / hyperactivity disorder.

### Stimulus presentation

Stimuli were presented using the Psychophysics Toolbox (Brainard, 1997; Kleiner et al., 2007; Pelli, 1997) on a TFT-LCD display (width: 33.7 cm, height: 27.0 cm, 1280 × 1024, refresh rate 120 Hz; Tobii Technology, Danderdyd, Sweden). Viewing distance from the screen was fixed using a chin rest (60 cm), and all testing took place in a darkened room.

### Procedure: Experiment 3

**Figure 1:**
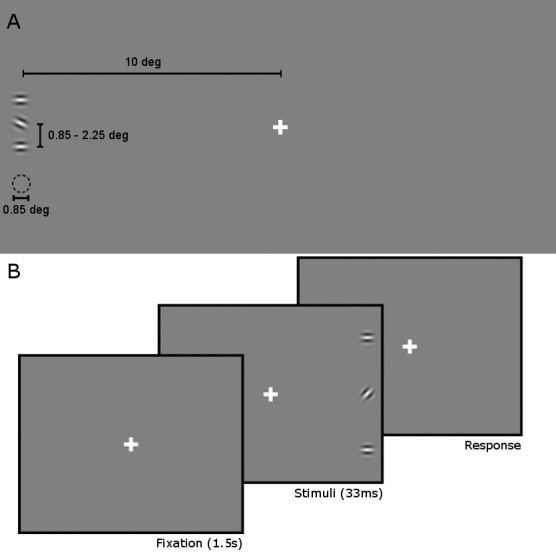
Display layout (A) and trial time course (B). Images not to scale. (A) The target was presented 10° of visual angle from the fixation cross. It was flanked by two identical, horizontal Gabor patches. To measure the spatial range of the crowding effect, target–flanker distance varied on each trial (0.85–2.25°). Only the closest target–flanker distance (0.85°) is shown. (B) Stimulus presentation lasted 33 ms, and was preceded by 1.5 s of fixation time (the intertrial interval). Response time was left to the participant, but could last a maximum of 5 s, after which the trial was counted as an error.

On each trial, participants fixated on a cross (white, diameter: 0.3°) in the center of the screen and reported the orientation of a peripheral Gabor target (Radius: 0.85°; Gaussian envelope: standard deviation of 0.15°, maximum 100%; Sinusoidal modulation: 3.5 c/°; Duration: 33.3 ms), presented on the horizontal meridian of the screen, 10° either to the left or right of fixation (Figure 1a). The target was tilted either clockwise (CW) or counterclockwise (CCW) from horizontal, and participants were asked to report the direction of tilt by pressing either the left-button (CW) or the up-button (CCW) on a standard keyboard. The angle of the target was set to each participants' 75% correct detection, obtained through a standard thresholding procedure after thorough practice with the task (see *Practice and Thresholding*).

To measure the spatial extent of crowding, this orientation discrimination task was embedded in a standard flanker paradigm. On each trial, the target was presented in the context of two flankers (Radius: 0.85°; Gaussian envelope: standard deviation of 0.15°, maximum 100%; Sinusoidal modulation: 3.5 c/°; Duration: 33.3 ms), one directly above and one below the target. These flankers had no tilt (horizontal orientation), and were presented at one of six distances from the target on each trial (0.85°, 1.05°, 1.25°, 1.45°, 1.85°, and 2.25°; Figure 1). Participants were allowed 5 s to respond to each trial, and a fixation period of 1.5 s began each trial. Participants were instructed to respond as soon as they knew the answer. Trials were presented using the method of constant stimuli (18 trials at each distance and screen side, 216 trials total), and screen side and target tilt were counterbalanced across trials. Response time and accuracy were recorded on each trial. During all experiments, subjects were instructed to maintain fixation throughout the experiment, and fixation accuracy was monitored throughout the experiment (see *Eye tracking and gaze analysis* in the following material).

### Procedure: Practice

Three blocks of practice trials preceded the experimental stage. In the first block (20 trials), the stimuli were presented on the screen until the participant responded. In the second block (96 trials), presentation time was gradually reduced from 300 ms to 33 ms. In the third block (96 trials), stimuli were presented for 33 ms. The target angle was randomly chosen on each practice trial (min = 20, max = 45°), and the target–flanker distance was randomly picked out of the six possible distances (Figure 1). To ensure participants performed above chance, participants were required to achieve at least 60% overall accuracy during these practice blocks to proceed to the full experiment, otherwise they did not proceed with the study. Given 96 practice trials, 60% is the smallest accuracy level that is significantly different from chance [*χ*^2^(1, *N* = 96) *=* 4.167*, p =* 0.041].

### Procedure: Thresholding experiments (Experiments 1–2)

After practice, we determined two perceptual thresholds using a staircase procedure: (a) the tilt of the target at which participants achieved 75% performance accuracy without flankers, (b) participants 75% discrimination thresholds under crowding at the closest target–flanker distance. The target was initially presented at 45° of rotation. With each trial, the angle of rotation was reduced by one step size if the response was correct, and increased by three step sizes if the response was incorrect. For the first five reversals, the step size was 7.5% of the previous orientation. Then, for 15 reversals, the step size was 2.5% of the previous orientation. The average of the last seven reversals was taken as the 75% threshold. Each individual's 75% correct crowded orientation discrimination threshold from Experiment 2 was used as the target tilt in Experiment 3. Crowding effects are diminished when the target is not perceptually grouped with the surrounding flankers (Livne & Sagi, 2010, 2011; Saarela, Sayim, Westheimer, & Herzog, 2009; Yeotikar, Khuu, Asper, & Suttle, 2011). To avoid breaks in perceptual grouping known to occur when targets and flankers are orthogonal (Livne & Sagi, 2011; Yeotikar et al., 2011), we excluded participants whose threshold was closer to being orthogonal than parallel (above 45°, *N* = 6, 5 with ASC).

### Performance analysis: Experiment 3

To analyze performance in the six distance conditions, we first discarded all trials in which responses were faster than 150 ms, or more than two standard deviations outside of their mean reaction time. We also discarded trials on which fixation was broken (see *Eye tracking and gaze analysis* below*)*. In order to estimate the critical distance, the target–flanker distance at which performance reaches 90% of the plateau value, we also fit exponential curves for each subject's data based on previous literature (Yeshurun & Rashal, 2010) using the following equation:

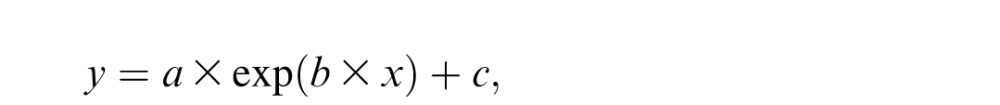

,
where *x* refers to the target–flanker distance, with the constraints that *a* and *b* had to be negative, and *c* had to fall between 0.5 and 1. −0.2, −2, and 0.95 were chosen as the starting values for *a*, *b*, and *c*, respectively. This ensured that the curve rose with increasing distance, and plateaued to a value between 50% and 100% performance (the lower and upper bound). The critical distance was therefore calculated as:

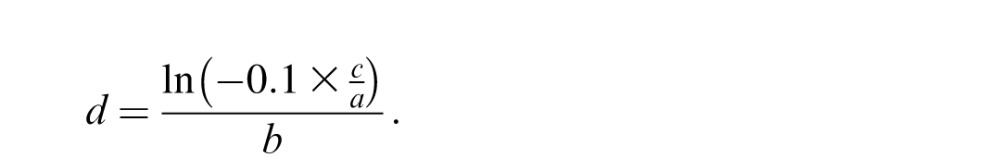

.



### Eye tracking and gaze analysis

Eye tracking was performed using a Tobii T120 eye tracker, via the Tobii MATLAB SDK (Tobii Technology, sampling rate 120 Hz, spatial resolution 0.5°). Participants successfully completed a nine-point calibration routine that was repeated if the eye tracker detected gaze position more than 0.92° away from the actual gaze position. Tracking was performed throughout the whole experiment. Fixation data from the left eye were analyzed starting 250 ms before stimulus onset until stimulus offset using custom MATLAB analysis scripts. Data from time points during which the eye-tracker did not receive input from the eye (e.g., blinks) were removed from the analysis. We excluded trials in which it was impossible to determine eye position due to lack of sampling, and trials in which the median eye position was more than 2° from the fixation point. We then excluded participants for whom more than 50% of trials in any condition were missing due to lack of fixation or eye tracking data (*n* = 7, 2 with ASC). This did not affect the outcome of any statistical tests used.

## Results

### Experiment 1: No impairment of orientation discrimination in ASC

**Figure 2:**
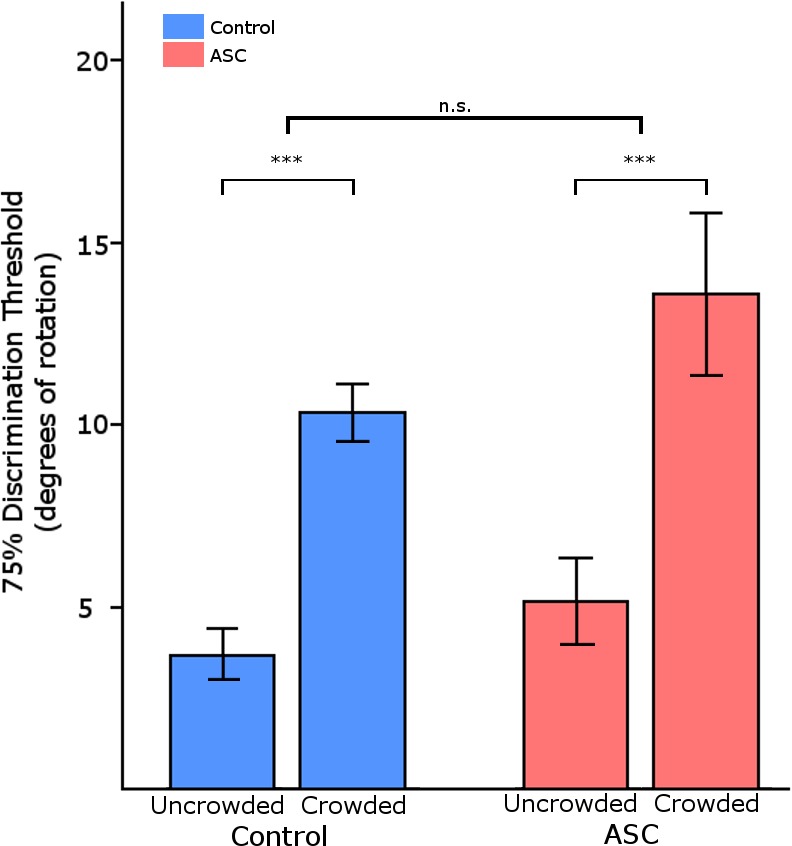
75% discrimination thresholds in Experiment 1 (Uncrowded) and 2 (Crowded). Error bars represent one standard error of the mean. The crowding effect was significant in both groups (***, *p* < 0.001). This effect was equally strong in both groups, and there were no group differences overall.

We first aimed to characterize peripheral orientation discrimination in individuals with and without ASC. We used a staircase procedure to identify the angle of rotation at which participants were able to identify the orientation correctly with 75% accuracy. We found no differences between the orientation discrimination thresholds of participants with and without ASC [U(18, 17) = 100, *p* < 0.247, mean ASC: 5.1°, mean Control: 3.7°, Cohen's *d* = 0.36, Figure 2]. This demonstrates that orientation discrimination in peripheral vision is comparable between individuals with and without ASC.

### Experiment 2: Equivalent maximum effect of crowding

Next, we aimed to characterize the magnitude of crowding in individuals with and without ASC. We therefore tested orientation 75% orientation discrimination thresholds used the same staircase procedure as before, but this time under crowded conditions (using the closest target–flanker distance in Experiment 3, 0.85°). As expected, both groups evidenced a significant increase in thresholds during crowding: a 2 × 2 ANOVA with Flanker Presence as within group factor and Diagnosis as between group factor revealed a main effect of Flanker Presence, *F*(1, 33) = 29.814, *p* < 0.001, Figure 2. However, there was no interaction between Group and Flanker Presence, *F*(1, 33) = 0.285, *p* < 0.597, mean ASC: 13.2°, mean Control: 10.35°, indicating that the magnitude of this effect was comparable between the two groups. This shows that the addition of flankers introduced equal levels of difficulty to the task for the two groups.

### Experiment 3: Comparable performance in both groups

**Figure 3:**
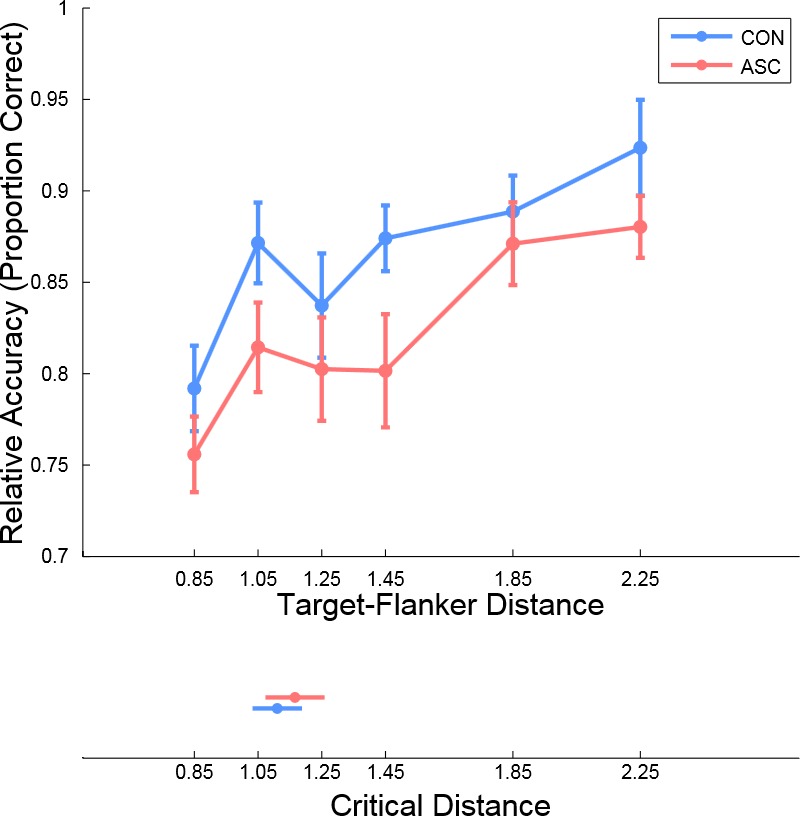
Results from Experiment 3. Mean accuracy of the two groups at the six target–flanker distances and the estimated critical distance for each group (inset). Error bars represent one standard error of the mean. Performance increased significantly with increasing target–flanker distance in both groups. The rate of this change was comparable between the two groups, and there was no overall difference in performance.

Having established comparable orientation discrimination and crowding effects at close target–flanker distances in ASC, we next sought to investigate whether the spatial extent of crowding differs between individuals with and without ASC. We therefore measured performance at six target–flanker distances using the method of constant stimuli. In Experiment 3, the target tilt was set to the individual's 75% accuracy threshold at the closest distance (see *Experiment 2*). We analyzed mean accuracy and median reaction time with separate 2 × 6 repeated-measures ANOVAs, using Distance as the within-subject factor and Diagnosis as the between-subject factor (Figure 3).

As predicted, performance accuracy increased with flanker distance in both groups, as evidenced by a significant main effect of Distance, *F*(5, 165) = 14.949, *p* < 0.001, Figure 3. This finding demonstrates that the effect of crowding on visual performance decreased with target–flanker distance. However, the magnitude of this effect was comparable between the two groups: No interaction between Diagnosis and Target–Flanker Distance was observed, *F*(5, 165) = 0.667, *p* < 0.626, *η_p_*^2^ = 0.02. Finally, no main effect of Diagnosis was observed, *F*(1, 33) = 2.782, *p* < 0.105, *η_p_*^2^ = 0.077, indicating overall comparable performance between the two groups. In sum, individuals with and without ASC demonstrated a comparable release from the crowding effect on visual performance with increasing target–flanker distances.

Reaction times in both groups were comparable [main effect of Diagnosis: *F*(1, 33) = 1.683, *p* < 0.204], and were not significantly modulated by Distance [main effect of Distance: *F*(5, 165) = 2.090, *p* < 0.086]. No interactions involving Diagnosis were observed [Diagnosis × Target–Flanker Distance, *F*(5, 165) = 0.961, *p* < 0.431]. To explore potential group differences in performance while accounting for any potential speed–accuracy trade-offs, we also analyzed performance using a combined metric of accuracy and response time (inverse efficiency scores: −1 × median reaction time / accuracy). Results of this analysis revealed a strong modulation of efficiency by Distance, *F*(5, 165) = 10.815, *p* < 0.001, but no interactions or main effects involving Diagnosis were observed (all *p* > 0.11). In sum, individuals with and without ASC evidenced a comparable magnitude of crowding, which decreased with target–flanker distance at a comparable rate.

### Experiment 3: Equivalent critical distances in both groups

To test whether the spatial extent of crowding differed between individuals with and without ASC, we calculated each individual's critical distance, the target–flanker distance at which performance reaches 90% of the plateau value. To do so, we fit an inverse exponential curve to each participant's data. This curve allowed us to estimate plateau performance for the two groups. We found no significant difference in this plateau value between groups [mean Control: 90.6% ± 8.3%, mean ASC: 87.0% ± 7.3%, *t*(32) = 1.361, *p* = 0.183], or between the estimated plateau value and the value achieved at the target–flanker distance of 2.25° [mean Control: 1.7% ± 4.4%, mean ASC: 1.0% ± 4.1%, *t*(32) = 0.462, *p* = 0.647]. The critical distance was not significantly different between the two groups, U(17, 18) = 109, *p* < 0.63; mean ASC: 1.11° ± 0.32°, mean Control: 1.17° ± 0.38°; Cohen's *d* = 0.17, indicating that crowding takes place across a similar spatial extent between participants with and without autism. Finally, no correlations between the critical distance and measures of symptomatology were observed (AQ: *p* < 0.928, ADOS: *p* < 0.940).

### Equivalent fixation stability in both groups

We analyzed gaze data to determine stability of fixation in both groups. We excluded trials in which it was impossible to determine eye position due to lack of sampling, and trials in which the median eye position was more than 2° of visual angle from the fixation point (excluded trials: 17% CON group, 23% ASC group). Participants were excluded for poor gaze stability if more than 50% of trials were excluded at any experimental distance, to ensure accuracy could be adequately estimated at that distance. There was no difference in the amount of trials excluded in the two groups, *t*(33) = 0.703, *p* < 0.487, indicating that differences in fixation patterns between groups are unlikely to influence our results.

## Discussion

We aimed to test the magnitude and spatial extent of crowding in individuals with autism spectrum conditions during a peripheral orientation discrimination task. We demonstrate typical orientation discrimination thresholds in ASC, which are affected by crowding to a similar degree as in controls. Further, we find that the spatial extent of crowding is typical in ASC: Crowding effects decline with increasing target–flanker distances at a comparable rate as in controls. These findings suggest that the bias towards local visual processing often reported in autism is unlikely to stem from a reduction in visual crowding.

Previous findings of typical contrast sensitivity (Koh, Milne, & Dobkins, 2010) and visual acuity (Albrecht et al., 2014; Bölte et al., 2012; Kéïta et al., 2010) in individuals with ASC suggest that many measures of low-level perception are typical in the condition. Our findings add to this pattern of results, indicating that this low-level measure of visual function, cardinal orientation discrimination, is also typical in autism. Importantly, cardinal orientation thresholds are known to exhibit a narrow dynamic range relative to oblique thresholds (Appelle, 1972) and rely on different neural mechanisms (Li, Peterson, & Freeman, 2003). One recent investigation found superior oblique orientation discrimination in typical individuals with higher autistic traits (Dickinson, Jones, & Milne, 2014). Therefore, future work should compare oblique and horizontal orientation discrimination in individuals with ASC.

Despite many reports of typical low-level visual function in ASC, numerous reports of atypical spatial processing of local visual information exist in the literature, especially in the context of cluttered visual displays (Almeida, Dickinson, Maybery, Badcock, & Badcock, 2012; O'Riordan et al., 2001; Plaisted et al., 1998). Previous research has also documented a sharper fall-off in visual performance with distance from a cued location in ASC, which strongly predicted autistic symptomatology (Robertson et al., 2013). However, both of these findings could arise from either a sharper allocation of attention in space (Robertson et al., 2013; Ronconi, Gori, Ruffino, Molteni, & Facoetti, 2013), a difference in the receptive field size of individuals with ASC (Schwarzkopf, Anderson, Haas, White, & Rees, 2014), or alterations at both levels of visual processing.

Our findings suggest that crowding is unlikely to be a component mechanism of the putative superior spatial range of visual processing in ASC. In this study, we specifically used Gabor targets because of their relative simplicity, predictions for a pooling model of crowding (Parkes et al., 2001), and relative insusceptibility to effects of attention load during crowding (Dakin, Bex, Cass, & Watt, 2009). We cannot rule out that attention may contribute to the effects observed here, as spatial attention is known to reduce the critical distance during crowding (Yeshurun & Rashal, 2010), although recent reports indicate a typical effect of attention on the critical distance during crowding among letter stimuli in ASC (Grubb et al., 2013).

It is unlikely that we failed to reject the null hypothesis of our study—that crowding is comparable between individuals with and without autism—by chance. Our experimental paradigm produced large and reliable effects of Target–Flanker distance on crowding in both groups, indicating that our manipulation was successful. Further, the variability in this effect within each group was larger than the variability between groups (*F*-value for a Group × Target–Flanker Distance interaction was < 1), suggesting that the group difference in crowding is smaller than even individual differences in crowding.

Our findings add to mounting evidence for typical crowding effects in ASC using a wide range of visual stimuli. Two previous papers reported no difference between participants with and without ASC using multifeature stimuli (letters and objects, Constable et al., 2010; Grubb et al., 2013). One paper reported a post-hoc difference between an ASC and Control group in crowding at close distances using object stimuli, but this finding was not supported by a significant Group × Distance interaction in the study (Kéïta et al., 2010). Although one paper reported a striking absence of crowding in ASC using Gabor patches (Baldassi et al., 2009), methodological differences may explain these apparently discrepant results. Baldassi et al. reported crowding effects as normalized by raw orientation discrimination thresholds, which was reported to be worse in their ASC group. It is therefore difficult to establish whether their findings are driven by worse orientation discrimination in ASC. Additionally, it is important to note that the only previous study to control for eye movements found typical crowding effects in ASC (Grubb et al., 2013). Together with previous reports, our findings of typical crowding effects in ASC using orientation stimuli suggest that the reported abnormalities in spatial processing observed in ASC are unlikely to arise from atypical crowding in the condition.

## Conclusions

In conclusion, we provide evidence for typical crowding in the peripheral visual fields of individuals with autism spectrum conditions. We find that the magnitude and spatial extent of crowding during a simple orientation discrimination paradigm in individuals with ASC are strong and indistinguishable from age- and IQ-matched controls. We also provide evidence of typical cardinal orientation discrimination in the condition. In sum, these results suggest that crowding is unlikely to drive the atypical spatial processing of visual information reported in ASC.

## Supplementary Material


