# Supplementary figures and images for: Typical magnitude and spatial extent of crowding in autism
Source: J Vis. 2016 Mar 21;16(5):17. doi: 10.1167/16.5.17 (PMC4811004; doi:10.1167/16.5.17)

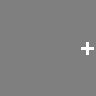

Supplement: Supplementary file 1 [file i1534-7362-16-5-17-icon.gif]
